# Xuebijing Injection Combined with Antibiotics for the Treatment of Spontaneous Bacterial Peritonitis in Liver Cirrhosis: A Meta-Analysis

**DOI:** 10.1155/2018/2989846

**Published:** 2018-03-19

**Authors:** Dan Han, Ran Wang, Yang Yu, Mingyu Sun, Rolf Teschke, Fernando Gomes Romeiro, Andrea Mancuso, Tingxue Song, Zhong Peng, Bing Han, Xinmiao Zhou, Wenchun Bao, Qianqian Li, Kexin Zheng, Yingying Li, Zhaohui Bai, Xiaozhong Guo, Xingshun Qi

**Affiliations:** ^1^Liver Cirrhosis Study Group, Department of Gastroenterology, General Hospital of Shenyang Military Area, Shenyang, China; ^2^Postgraduate College, Liaoning University of Traditional Chinese Medicine, Shenyang, China; ^3^Institute of Liver Diseases, Shuguang Hospital Affiliated to Shanghai University of Traditional Chinese Medicine, Shanghai, China; ^4^Department of Internal Medicine II, Division of Gastroenterology and Hepatology, Klinikum Hanau, Hanau, Germany; ^5^Teaching Hospital of the Medical Faculty, Goethe University, Frankfurt/Main, Germany; ^6^Department of Internal Medicine, Botucatu Medical School, Universidade Estadual Paulista (UNESP), Av. Prof. Mário Rubens Guimarães Montenegro, s/n, Distrito de Rubião Jr., 18 608 917 Botucatu, SP, Brazil; ^7^Internal Medicine at ARNAS Civico, Palermo, Italy; ^8^Postgraduate College, Dalian Medical University, Dalian, China; ^9^Postgraduate College, Jinzhou Medical University, Jinzhou, China; ^10^Postgraduate College, Shenyang Pharmaceutical University, Shenyang, China

## Abstract

**Background and Aim:**

Spontaneous bacterial peritonitis (SBP) is one of the most common complications of liver cirrhosis. Antibiotics are the main treatment regimen of SBP. Traditional Chinese medicine Xuebijing injection has been used in such patients. Our study aimed to overview the efficacy of Xuebijing injection combined with antibiotics for the treatment of SBP.

**Method:**

We searched the PubMed, Embase, China National Knowledge Infrastructure, VIP, and Wanfang databases. The search items included “Xuebijing”, “peritonitis”, “liver cirrhosis”, and “random” to identify all relevant randomized controlled trials (RCTs). The Cochrane risk of bias tool was used to assess the study quality. The odd ratios (ORs) with 95% confidence intervals (CIs) were calculated by using a random-effect model. Heterogeneity was also calculated.

**Results:**

A total of 9 RCTs were included. The study quality was unsatisfied. The overall (OR = 2.95, 95% CI = 1.97–4.42, *p* < 0.00001) and complete (OR = 2.18, 95% CI = 1.57–3.04, *p* < 0.00001) responses were significantly higher in the Xuebijing injection combined with antibiotics group than the antibiotics alone group. The incidence of cirrhosis related complications, including hepatic encephalopathy and hepatorenal syndrome, was lower in the Xuebijing injection combined with antibiotics group than the antibiotics alone group. No significant heterogeneity was observed among studies.

**Conclusion:**

Additional use of Xuebijing injection may improve the efficacy of antibiotics for the treatment of SBP in liver cirrhosis. However, due to a low level of current evidence, we did not establish any recommendation regarding the use of Xuebijing injection for the treatment of SBP.

## 1. Introduction

Patients with liver cirrhosis are prone to bacterial infection, principally including spontaneous bacterial peritonitis (SBP) [1] and urinary tract infection [2]. SBP is a common and serious complication in cirrhotic patients with ascites [3]. The incidence of SBP in decompensated cirrhosis is 10%–30% [4], and the mortality is 10%–50% [5, 6]. Mostly pathogenic bacteria that cause SBP come from the gut, of which the most common is* Escherichia coli* [7]. SBP should be actively managed to reduce its related morbidity and mortality. The third-generation cephalosporins are the first-line choice of therapy for SBP [3, 8]. Levofloxacin and amoxicillin-clavulanic acid may be alternatives in cirrhotic patients [9].

A previous systematic review by our study group showed that Xuebijing injection was the most commonly used traditional Chinese medicine drug for the treatment of SBP [10]. Xuebijing injection is primarily based on the ancient blood-regulating formula proposed by Dr. Qingren Wang, a famous traditional Chinese medicine physician. It includes Honghua, Red Peony Root, Chuanxiong Root, Danshen Root, and angelica sinensis [11], which can promote blood circulation and remove blood stasis. Nowadays, Xuebijing injection has been widely used for the treatment of sepsis [12] and acute pancreatitis [13] in China. The efficacy and safety of Xuebijing injection for the treatment of SBP in liver cirrhosis remains unclear. Herein, our study aimed to further explore this issue using the method of systematic review and meta-analysis.

## 2. Methods

### 2.1. Registration

The registration number of PROSPERO was CRD42017070992.

### 2.2. Search Strategy

PubMed, Embase, China National Knowledge Infrastructure (CNKI), VIP, and Wanfang databases were searched from inception to July 2017. The search items were “Xuebijing” AND “peritonitis” AND “liver cirrhosis” AND “random”.

### 2.3. Inclusion and Exclusion Criteria

All randomized controlled trials (RCTs) regarding Xuebijing injection for the treatment of SBP in patients with cirrhosis were included. The Xuebijing injection group should be patients who received Xuebijing injection combined with antibiotics. The control group should be patients who received antibiotics alone. Exclusion criteria were as follows: (1) duplicates; (2) systematic reviews, and/or meta-analyses; (3) catalogue, indexes, and conferences; and (4) irrelevant topics. There was no limit on publication status or language.

### 2.4. Data Extraction

Data were extracted as follows: (1) general information: title, author's information, year of publication, and region; (2) characteristics of studies: study design, objective of study, and method of intervention; and (3) outcomes: overall response, complete response, no response, incidence of cirrhosis related complications, and drug related adverse events.

Definitions of complete response were based on the included studies. In detail, response was assessed according to the change in clinical symptoms, serum white blood cell, white blood cell in ascites, polymorphonuclear leukocytes in ascites, and/or bacterial culture in ascites.

### 2.5. Risk of Bias Assessment

Cochrane risk of bias tool was used to assess the study quality. It includes (1) random sequence generation, (2) allocation concealment, (3) blinding of participants and personnel, blinding of outcome assessment, (4) incomplete outcome data, (5) selective reporting, and (6) other bias.

### 2.6. Data Analysis

The statistical analysis was performed using Review Manager 5.2. Pooled data were analyzed by using odds ratios (ORs) with 95% confidence intervals (CIs). The heterogeneity was evaluated by Cochran's *Q* test and *I*^2^. *p* < 0.1 or *I*^2^ > 50% represents a significant heterogeneity. A random-effect model was used. Subgroup analyses were conducted based on the type of antibiotics (cephalosporins or non-cephalosporins antibiotics). The funnel plots were used to assess the presence of publication bias. A *p* value of <0.05 was considered statistically significant.

## 3. Results

### 3.1. Selection of Studies

**Figure 1:**
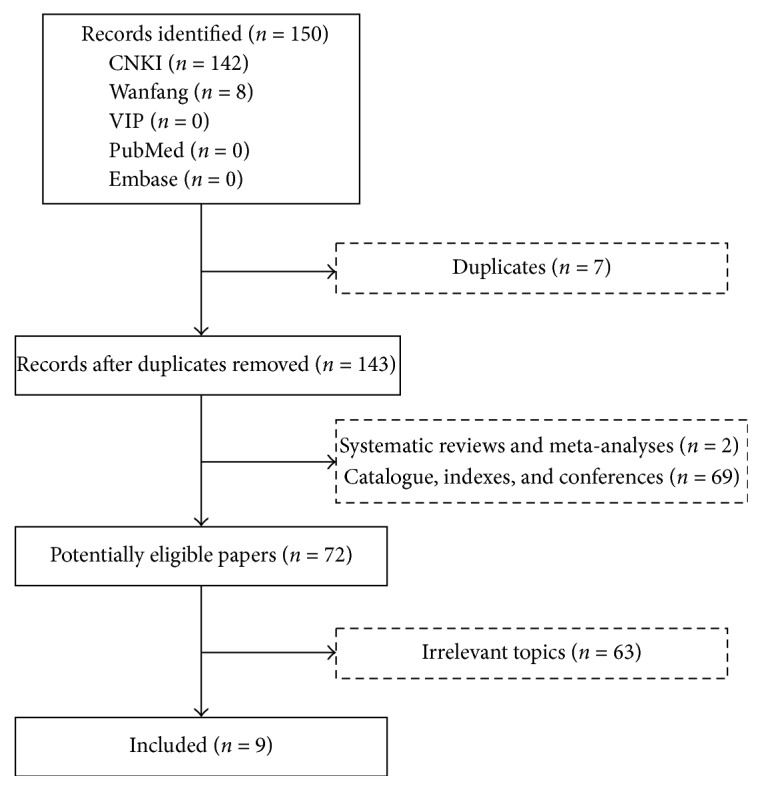
Flowchart of study selection.

Overall, 150 studies were initially identified (142 studies from CNKI and 8 studies from Wanfang database). Among them, 9 studies [14–22] were finally included (Figure 1). The characteristics of these studies were shown in Tables 1 and 2.

### 3.2. Risk of Bias

Regarding sequence generation, 2 studies had a low risk of bias and the remaining 7 studies had an unclear risk. Regarding incomplete outcome data and selective report, all studies had a low risk of bias. Regarding allocation concealment, blinding of participants and personnel, blinding of outcome assessment, and other bias, all studies had an unclear risk of bias (Supplementary Figure 1).

### 3.3. Overall Response

**Figure 2:**
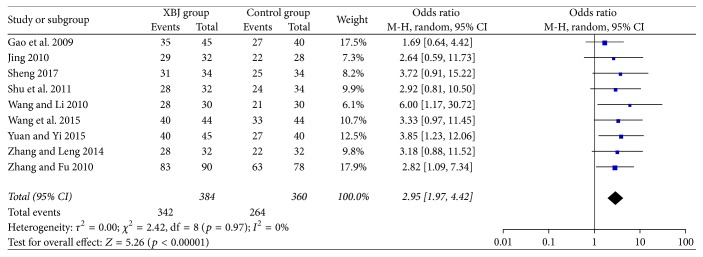
Comparison of overall response between Xuebijing injection and control groups.

All included studies reported the data regarding overall response. The Xuebijing injection group included 384 patients, of whom 342 patients (89.1%) had an overall response. The control group included 360 patients, of whom 264 (73.3%) patients had an overall response. The Xuebijing injection group had a significantly higher overall response than the control group (OR = 2.95, 95% CI = 1.97–4.42, *p* < 0.00001) (Figure 2). There was no significant heterogeneity (*I*^2^ = 0%; *p* = 0.97).

The Xuebijing injection group had a significantly higher overall response than the control group in the subgroup analyses of cephalosporin (OR = 2.84, 95% CI = 1.81–4.45, *p* < 0.00001) and non-cephalosporin antibiotics (OR = 3.50, 95% CI = 1.38–8.84, *p* = 0.008) (Supplementary Figure 2). There was no significant heterogeneity.

### 3.4. Complete Response

**Figure 3:**
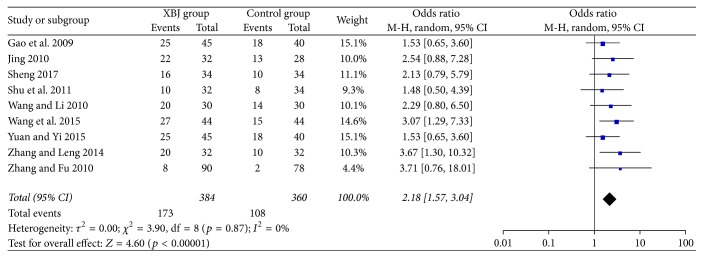
Comparison of complete response between Xuebijing injection and control groups.

All included studies reported the data regarding complete response. The Xuebijing injection group included 384 patients, of whom 173 patients (45.1%) had a complete response. The control group included 360 patients, of whom 108 patients (30.0%) had a complete response. The Xuebijing injection group had a significantly higher complete response than the control group (OR = 2.81, 95% CI = 1.57–3.04, *p* < 0.00001) (Figure 3). There was no significant heterogeneity (*I*^2^ = 0%; *p* = 0.87).

The Xuebijing injection group had a significantly higher complete response than the control group in the subgroup analyses of cephalosporin (OR = 2.05, 95% CI = 1.39–3.01, *p* = 0.0003) and non-cephalosporin antibiotics (OR = 2.62, CI = 1.36–5.06, *p* = 0.004) (Supplementary Figure 3). There was no significant heterogeneity.

### 3.5. No Response

**Figure 4:**
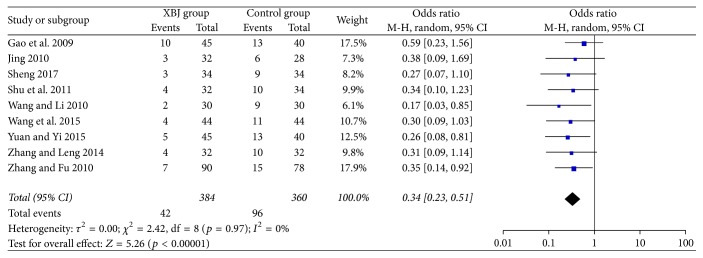
Comparison of no response between Xuebijing injection and control groups.

All included studies reported the data regarding no response. The Xuebijing injection group included 384 patients, of whom 42 patients (10.9%) had no response. The control group included 360 patients, of whom 96 patients (26.7%) had no response. No response was significantly lower in the Xuebijing injection group than the control group (OR = 0.34, 95% CI = 0.23–0.51, *p* < 0.00001) (Figure 4). There was no significant heterogeneity (*I*^2^ = 0%; *p* = 0.97).

No response was significantly lower in the Xuebijing injection group than the control group in the subgroup analyses of cephalosporin (OR = 0.35, 95% CI = 0.22–0.55, *p* < 0.00001) and non-cephalosporin antibiotics (OR = 0.29, CI = 0.11–0.72, *p* = 0.008) (Supplementary Figure 4). There was no significant heterogeneity.

### 3.6. Cirrhosis Related Complications

The incidence of cirrhosis related complications, including gastrointestinal bleeding, hepatic encephalopathy, and hepatorenal syndrome, was reported in 3 studies. The incidence of septic shock was reported in 2 studies (Table 3).

The Xuebijing injection group had a lower incidence of gastrointestinal bleeding than the control group, but no significant difference was observed between them (OR = 0.63, 95% CI = 0.18–2.16, *p* = 0.46) (Supplementary Figure 5). There was no significant heterogeneity (*I*^2^ = 0%; *p* = 0.61).

The Xuebijing injection group had a significantly lower incidence of hepatic encephalopathy than the control group (OR = 0.19, 95% CI = 0.08–0.47, *p* = 0.0004) (Supplementary Figure 6). There was no significant heterogeneity (*I*^2^ = 0%; *p* = 1.00).

The Xuebijing injection group had a significantly lower incidence of hepatorenal syndrome than the control group (OR = 0.38, 95% CI = 0.21–0.70, *p* = 0.002) (Supplementary Figure 7). There was no significant heterogeneity (*I*^2^ = 0%; *p* = 0.76).

The Xuebijing injection group had a lower incidence of septic shock than the control group, almost achieving the significance level (OR = 0.20, 95% CI = 0.04–0.99, *p* = 0.05) (Supplementary Figure 8). There was no significant heterogeneity (*I*^2^ = 0%; *p* = 1.00).

### 3.7. Drug Related Adverse Events

Only 2 studies reported the data regarding adverse events associated with Xuebijing injection. Shu et al. [17] did not find any adverse events. Wang et al. [19] found that 2 and 3 patients developed nausea and vomiting in the Xuebijing injection and the control groups, respectively. There was no significant difference in the incidence of adverse events between them.

## 4. Discussion

It has been reported that empirical antibiotics for the treatment of SBP may fail in almost 60% of patients and that 40% of patients with an initial response to empirical antibiotics may need to change the type of antibiotics [23]. Similarly, our study found that the complete response rate of antibiotics for SBP was 30.0%. By comparison, the complete response rate of Xuebijing injection combined with antibiotics for SBP was 45.1%. Thus, Xuebijing injection combined with antibiotics for the treatment of SBP might be more effective than antibiotics alone.

Evidence suggests that Xuebijing injection has antagonistic effects on endotoxin [24], inhibits the release of tumor necrosis factor [25], protects from the damage of endothelial cells [24], and promotes the recovery of immune function [26]. The China Food and Drug Administration approved the use of Xuebijing injection for the infection-induced systemic inflammatory response syndrome and multiple organ dysfunction syndrome in China. Xuebijing injection has been used for sepsis and pancreatitis in clinical practice [27]. We have to acknowledge that Xuebijing injection is not the main treatment strategy of SBP according to the international guideline. However, many studies from China have explored the efficacy of Xuebijing injection in the treatment of SBP. So we should further validate it by a meta-analysis of previous studies. Our study was the first meta-analysis to explore the efficacy of Xuebijing injection for the treatment of SBP.

We also conducted the subgroup analysis based on the type of antibiotics (i.e., cephalosporin and non-cephalosporin groups). The efficacy of Xuebijing injection remained regardless of type of antibiotics. Whether different types of antibiotics will affect the outcome of Xuebijing injection for the treatment of SBP needs to be further explored.

Except for SBP, cirrhosis is also prone to other complications, including gastrointestinal bleeding [28], hepatic encephalopathy [29], hepatorenal syndrome [30], and even septic shock [31]. We found that the use of Xuebijing injection could reduce the incidence of cirrhosis related complications in patients with SBP. Notably, bacterial infection is the major precipitating and aggravating factor for gastroesophageal variceal bleeding [32] and hepatic encephalopathy [33]. Thus, Xuebijing injection can reduce the risk of gastroesophageal variceal bleeding and hepatic encephalopathy by controlling bacterial infection. Until now, studies regarding Xuebijing injection for the treatment of cirrhosis related complications are lacking.

Several studies found that the primary adverse event of Xuebijing injection was immediate hypersensitivity reaction within 30 minutes and its main clinical manifestations were skin itch, chest distress, shortness of breath, palpitation, and blood pressure dropping [34–36]. Nevertheless, we found very few adverse events related to Xuebijing injection.

The limitations of our study are as follows. First, the quality of included studies was relatively poor, despite only RCTs were included. Second, the majority of studies did not give any information about the severity of liver disease. Third, the adverse events of Xuebijing injection in the treatment of SBP were rarely reported. Fourth, the follow-up duration was short. Fifth, the treatment strategy in the control group was often heterogeneous. Sixth, the diagnostic criteria for SBP were mostly inconsistent. Seventh, Xuebijing injection therapy was used only in China but not in other countries due to lack of this drug. All included studies had been conducted in China.

In conclusion, the efficacy of antibiotics for SBP in liver cirrhosis may be improved by additional use of Xuebijing injection. However, considering the low quality of included studies, large double-blinded randomized controlled trials are needed to prove whether Xuebijing injection can be used for the treatment of SBP. Additionally, Xuebijing injection, a TCM drug, is mainly used in China. We found a relatively high efficacy of Xuebijing injection and hoped that it might be extended to Western countries and even the whole world in the future. Future research should also explore the role of Xuebijing injection for the management of other liver cirrhosis related complications.

## Figures and Tables

**Table 1:** Characteristics of included studies.

First author (year)	Region	Journal	Study design	Groups	Interventions	Duration of treatment (days)	*p*
Sheng (2017)	Jiangxi	Medical Information	RCT	XBJ group	Antibiotics: imipenem and cilastatin sodium 0.5 g, 3 times/day; Xuebijing injection 50 ml, 1 time/day	7	<0.05
				Control group	Antibiotics: imipenem and cilastatin sodium 0.5 g, 3 times/day		

Yuan (2015)	Shandong	China Practical Medicine	RCT	XBJ group	Antibiotics: ceftriaxone sodium 2 g, 1 time/day; Xuebijing injection 50 ml, 2 times/day	10	<0.05
				Control group	Antibiotics: ceftriaxone sodium 2 g, 1 time/day		

Wang (2015)	Henan	Drugs & Clinic	RCT	XBJ group	Antibiotics: Imipenem and cilastatin sodium 0.5 g, 3 times/day; Xuebijing injection 50 ml, 1 times/day	7	<0.05
				Control group	Antibiotics: imipenem and cilastatin sodium 0.5 g, 3 times/day		

Zhang (2014)	Hunan	Medical Journal of West China	RCT	XBJ group	Antibiotics: ceftazidime 2 g, 1 time/day; Xuebijing injection 50 ml, 2 times/day	28	<0.05
				Control group	Antibiotics: ceftazidime 2 g, 1 time/day		

Shu (2011)	Guangdong	Chinese Journal of Integrated Traditional and Western Medicine on Digestion	RCT	XBJ group	Antibiotics: cephalosporin; Xuebijing injection 50 ml, 1 time/day	14	<0.05
				Control group	Antibiotics: cephalosporin		

Zhang (2010)	Guangdong	Progress in Modern Biomedicine	RCT	XBJ group	Antibiotics: ceftriaxone sodium; albumin Xuebijing injection 50 ml, 2 times/day	7	<0.05
				Control group	Antibiotics: ceftriaxone sodium albumin		

Wang (2010)	Tianjin	Journal of Clinical Pharmacy	RCT	XBJ group	Antibiotics: ceftriaxone 2 g, 1 times/day; Xuebijing injection 50 ml, 2 times/day	7	<0.05
				Control group	Antibiotics: ceftriaxone 2 g, 1 time/day		

Jing (2010)	Qinghai	Liaoning Journal of Traditional Chinese Medicine	RCT	XBJ group	Antibiotics: ceftazidime 2 g, 2 times/day; Xuebijing injection 60 ml, 1 time/day	7	<0.05
				Control group	Antibiotics: ceftazidime 2 g, 2 times/day		

Gao (2009)	Tianjin	Journal of Tianjin University of Traditional Chinese Medicine	RCT	XBJ group	Antibiotics: cephalosporin; albumin Xuebijing injection 50 ml, 2 times/day	10	>0.05
				Control group	Antibiotics: cephalosporin albumin		

RCT, randomized controlled trial; XBJ, Xuebijing injection.

**Table 2:** Characteristics of patients.

First author (year)	Number of pts	Etiology of liver disease	Sex (male/female) (*n*)	Age (mean)	Groups	Follow-up (days)	Overall response (*n*)	Complete response (*n*)	No response (*n*)	*p*
Sheng (2017)	34	NA	36/32	36.90	XBJ group	7	31	16	3	<0.05
	34	NA			Control group	7	25	10	9	

Yuan (2015)	45	NA	36/9	53.50	XBJ group	10	40	25	5	<0.05
	40	NA	32/8	52.30	Control group	10	27	18	13	

Wang (2015)	44	NA	23/21	37.85	XBJ group	7	40	27	4	<0.05
	44	NA	22/22	37.92	Control group	7	33	15	11	

Zhang (2014)	32	HBV 32	18/14	50.12	XBJ group	28	28	20	4	<0.05
	32		18/14	51.21	Control group	28	22	10	10	

Shu (2011)	32	HBV 49; HCV 7; alcohol 5; PBC 3; cryptogenic cirrhosis 2	54/12	33.92	XBJ group	14	28	10	4	<0.05
	34				Control group	14	24	8	10	

Zhang (2010)	90	Posthepatitic cirrhosis 73; alcohol 9; PBC 5; cryptogenic cirrhosis 3	62/28	47.20	XBJ group	7	83	8	7	<0.05
	78	Posthepatitic cirrhosis 67; alcohol 7; PBC 3; cryptogenic cirrhosis 1	61/17	48.10	Control group	7	63	2	15	

Wang (2010)	30	HBV 36; HCV 5; alcohol 14; PBC 2; unknown origin 3	47/13	51.38	XBJ group	7	28	20	2	<0.05
	30				Control group	7	21	14	9	

Jing (2010)	32	NA	23/9	49.30	XBJ group	7	29	22	3	<0.05
	28	NA	21/7	48.70	Control group	7	22	13	6	

Gao (2009)	45	NA	38/7	48.30	XBJ group	10	35	25	10	>0.05
	40	NA	35/5	46.20	Control group	10	27	18	13	

Pts, patients; XBJ, Xuebijing injection; HBV, hepatitis B virus; HCV, hepatitis C virus; PBC, primary biliary cirrhosis.

**Table 3:** Cirrhosis related complications.

First author (year)	Number of pts	Gastrointestinal bleeding	Hepatic encephalopathy	Hepatorenal syndrome	Septic shock
Gao (2009)	45	2	3	20	1
	40	1	11	25	4
Wang (2015)	44	1	0	0	NA
	44	2	2	1	NA
Yuan (2015)	45	2	3	15	1
	40	4	11	25	4

Pts, patients.

## Abbreviations

SBP: Spontaneous bacterial peritonitis
RCTs: Randomized controlled trials
ORs: Odds ratios
CIs: Confidence intervals
CNKI: China National Knowledge Infrastructure.

## Supplementary Materials

Supplementary 1 Supplementary Figure  1: risk of bias assessment.

Supplementary 2 Supplementary Figure  2: comparison of overall response between Xuebijing injection and control groups in subgroup analyses of cephalosporin and non-cephalosporin group.

Supplementary 3 Supplementary Figure  3: comparison of complete response between Xuebijing injection and control groups in subgroup analyses of cephalosporin and non-cephalosporin group.

Supplementary 4 Supplementary Figure  4: comparison of no response between Xuebijing injection and control groups in subgroup analyses of cephalosporin and non-cephalosporin group.

Supplementary 5 Supplementary Figure  5: comparison of the incidence of gastrointestinal bleeding between Xuebijing injection and control groups.

Supplementary 6 Supplementary Figure  6: comparison of the incidence of hepatic encephalopathy between Xuebijing injection and control groups.

Supplementary 7 Supplementary Figure  7: comparison of the incidence of hepatorenal syndrome between Xuebijing injection and control groups.

Supplementary 8 Supplementary Figure  8: comparison of the incidence of septic shock between Xuebijing injection and control groups.
